# Huge Primary Parasitic Leiomyoma in a Postmenopausal Lady: A Rare Presentation

**DOI:** 10.1155/2019/7683873

**Published:** 2019-04-01

**Authors:** Nkencho Osegi, E. Yilaiba Oku, C. Stanley Uwaezuoke, K. Timothy Alawode, S. Adeniyi Afolabi

**Affiliations:** ^1^Department of Obstetrics and Gynaecology, Federal Medical Centre, Yenagoa, Bayelsa State, Nigeria; ^2^Department of Surgery, Federal Medical Centre, Yenagoa, Bayelsa State, Nigeria; ^3^Department of Pathology, Federal Medical Centre, Yenagoa, Bayelsa State, Nigeria

## Abstract

Although uterine myomas are the most common benign tumours of the female pelvis in the reproductive age group, they rarely grow in menopausal women. Parasitic fibroids without prior history of laparoscopic myomectomy are even a rarer presentation particularly in menopausal women. The case presented is a 58-year-old grand-multiparous, menopausal lady with progressive abdominal swelling of three-year duration. She had excision of a huge parasitic fibroid attached to omentum. She had partial omentectomy, total abdominal hysterectomy, and bilateral salpingo-oophorectomy. The parasitic fibroid mass weighed 5.2kg and histopathology confirmed leiomyoma uteri with cystic degeneration and lymph nodes with reactive lymphoid hyperplasia. She had uneventful postoperative recovery and follow-up has so far been uneventful.

## 1. Introduction

Uterine fibroids are the most common tumours of the female genital tract in women within the reproductive age and it has been documented that they occur in over 70% of women by the onset of menopause [1]. Chief among the risk factors is race and it is more common in women of African ancestry. They are benign monoclonal tumours that arise from the uterine smooth muscle tissue. The initiating events for fibroid genesis remain speculative. The cells proliferate at a modest rate and their growth is dependent on the ovarian steroids oestrogen and progesterone and therefore most fibroids shrink after menopause [2].

Spontaneous parasitic fibroids are a rare subset of subserous fibroids that become adherent to other structures, obtain their blood supply from such structures, and usually do not retain vascular connection with the uterus [3]. Most reported cases are iatrogenic and are linked to unintentional seeding of the fragments during previous laparoscopic myomectomy using the morcellator [4–7]. The true incidence of parasitic fibroid is unknown, but some authors have reported an incidence of 0.9% in women with previous laparoscopic myomectomy [8]. We report a rare case of spontaneous parasitic leiomyoma occurring in a menopausal lady without history of previous operation.

## 2. Case Presentation

A 58-year-old menopausal woman, para 6+^0^, presented to the gynecology clinic with progressive abdominal swelling of three-year duration. There was no associated pain, vaginal bleeding/discharge, weight loss, gastrointestinal symptoms, or respiratory difficulties. She was eight years postmenopause. She was a known diabetic on oral medication and had been previously managed medically for uterine fibroids prior to menopause. She had no history of previous surgery. She had no family history of malignancy: breast, ovarian, or endometrial.

Examination revealed a markedly distended abdomen that moved with respiration. An abdominopelvic mass that extended to the xiphisternum was palpated. It was firm, nontender, and slightly mobile in the transverse plane. Other organs were not palpable and there was no demonstrable ascites.

Haemogram, electrolytes, urea, creatinine, and liver function tests were all normal. Serum Ca-125 was 21.3u/ml. Urinalysis showed no abnormality. Chest X-ray and ECG were normal. Abdominopelvic ultrasound scan showed a bulky nongravid anteverted uterus with multiple uterine fibroids with degenerative changes and a mid-line echo. A huge mass extending up to the epigastrium with mixed echogenicity and areas of cystic changes was seen. The liver, gall bladder, spleen, pancreas, and kidneys were grossly normal without focal mass lesion. No ascites was seen. The ovaries were not visualized.

**Figure 1:**
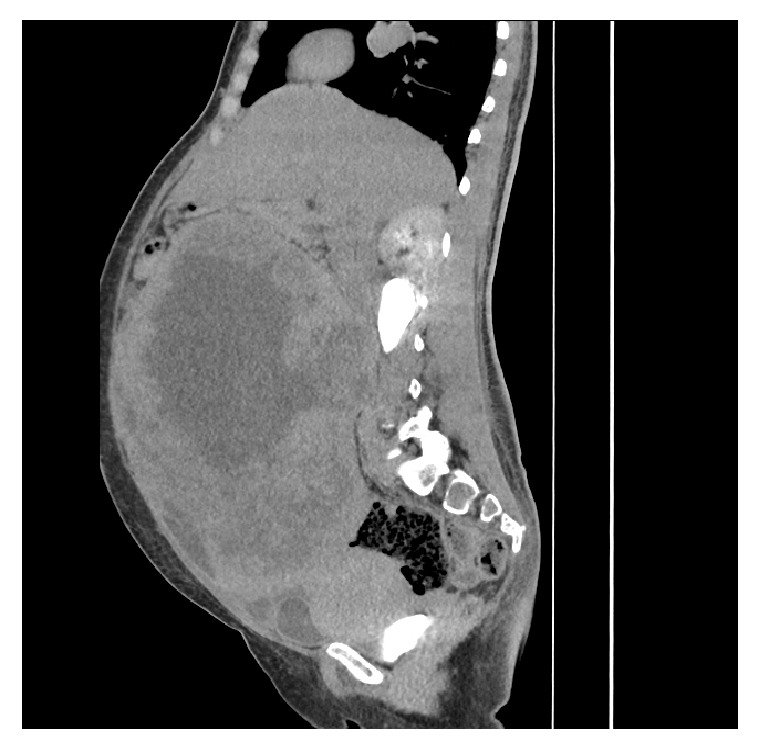
Presurgical evaluation showing the sagital slice of the abdominopelvic CT scan.

**Figure 2:**
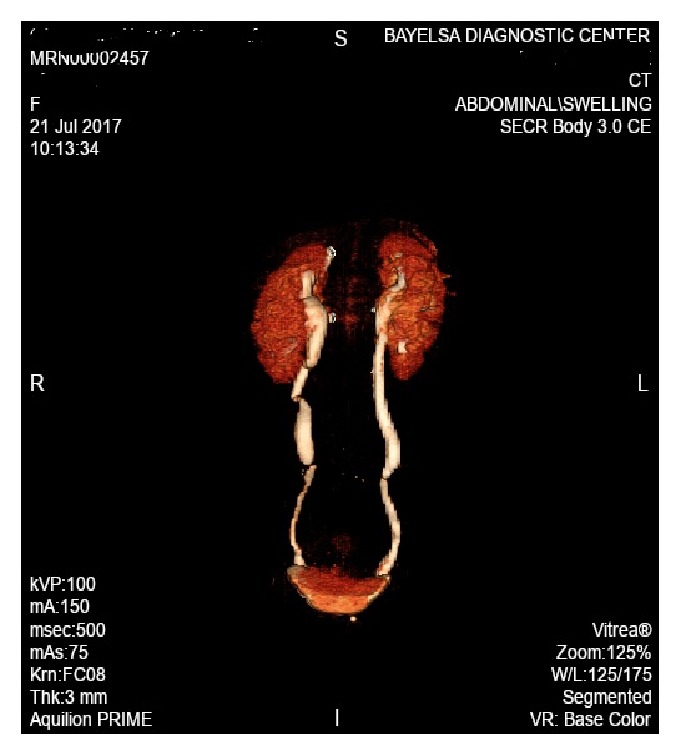
Presurgical evaluation outlining the kidneys, ureters, and bladder on contrast CT scan.

Abdominopelvic computerized tomographic scan with intravenous contrast (Figures 1 and 2) showed a large heterogenous predominantly necrotic mass measuring about 22.4 x 16 x 25cm extending from the mid-pelvis to the level of the epigastric region, mild bilateral hydroureteronephrosis due to compression of the mid-ureters by the abdominopelvic mass and mild ascites were noted.

**Figure 3:**
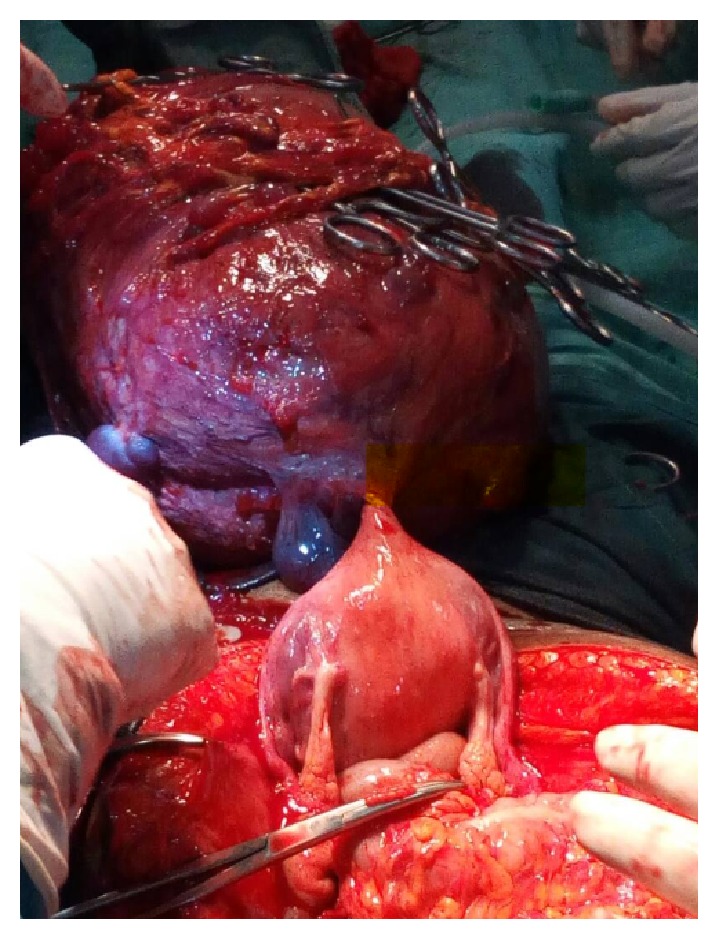
Intraoperative finding of a huge parasitic fibroid wrapped around and deriving its blood supply from the omentum.

She had exploratory laparotomy and findings at surgery were a huge mass with cystic and solid areas filling up the entire abdominal cavity up to the epigastrium pushing the bowels loops up and to the left (Figure 3). It derived its blood supply from the greater omentum to which it was attached with huge visible arteries and veins. Enlarged lymph nodes were noted in the gastrocolic ligament. The uterus had multiple myomas, but other structures (ovaries, fallopian tubes, liver, spleen, and intestines) were normal.

**Figure 4:**
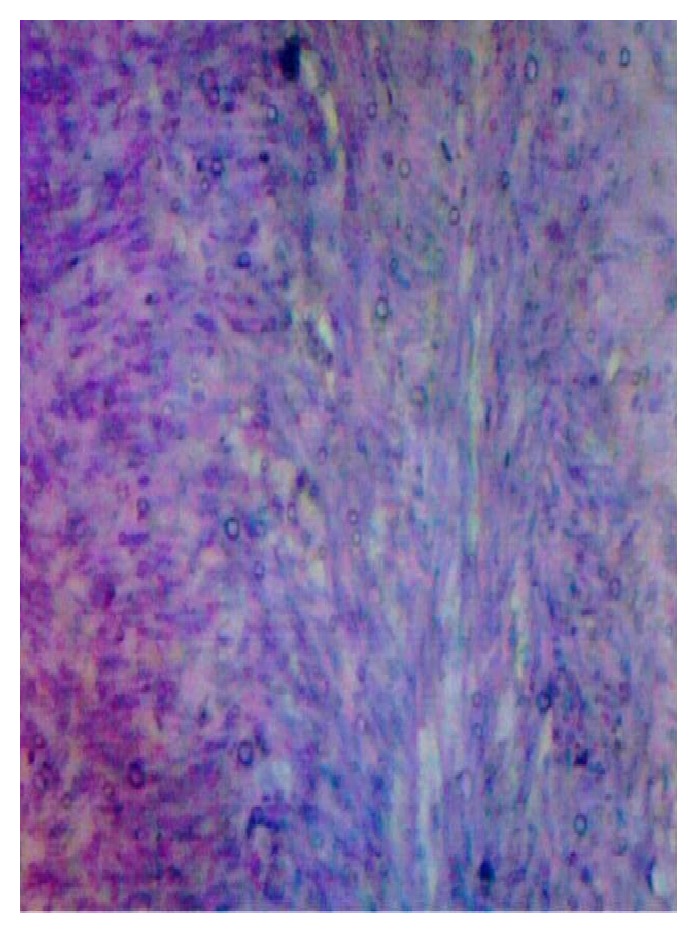
The microscopic slide of the histology.

She had excision of the huge parasitic fibroid, partial omentectomy, total abdominal hysterectomy and bilateral salpingo-oophorectomy. The histology report (Figure 4) confirmed leiomyoma with cystic degeneration and lymph nodes with reactive lymphoid hyperplasia.

## 3. Discussion

Very rarely, a subserous fibroid may detach from the uterus to attach and receive its blood supply from other structures in the abdomen. When this occurs in a woman without previous history of surgery as in the case presented, it is the considered opinion of the authors that this should be regarded as a primary or spontaneous parasitic fibroid. Primary parasitic fibroids are a rare variant of subserous fibroids that often pose diagnostic dilemma with diagnosis made mostly during surgery and/or following histopathology assessment of the tumour [9].

Secondary or iatrogenic parasitic fibroids are the more common form of parasitic fibroids and are seen as a complication of previous myomectomy particularly following laparoscopy using morcellator. The literature on parasitic fibroids is sparse and most reported cases have been linked to previous laparoscopic surgeries.

This case report of parasitic myoma occurred without history of prior abdominal procedure. Although this is not common, but few similar cases have been reported by others. Abdulwahid and associates reported parasitic fibroids in a 46-year-old lady without previous history of surgery [10]. This is similar to cases reported by Mushtaq [11] and Abdel-Gadir et al. [12]. Previous surgery (laparoscopy) may not be the most significant risk factor for development of parasitic fibroids as stated by many authors. Nehzat and colleagues [5] reported a series of parasitic fibroids and posited in their work that the greatest risk factor for development of parasitic myoma is the presence of uterine leiomyoma as found in the case presented.

Parasitic fibroids have been reported in premenopausal women without primary uterine fibroids; this however appears to be extremely rare in menopausal women as uterine fibroids seem to be a prerequisite for presence of parasitic fibroids in this group of women. The reason for this is unknown. This hypothesis is strengthened by Nappi and colleagues who reported parasitic leiomyoma coexisting with uterine leiomyoma in a 58-year-old postmenopausal woman similar to the case presented [13].

Although most parasitic myomas occur in the pelvis, index case was located on the greater omentum in the abdominal cavity which appears to be the most common source of blood supply. This finding is similar to what has been reported by other authors. More rare locations like the urethra and sigmoid colon have also been reported [14].

Clinical features of parasitic fibroids are usually nonspecific. Although they may be asymptomatic, symptoms when present depend on the size and location of the fibroids and are mostly due to pressure. Our patient presented with abdominal swelling. Management is usually surgical resection either via laparotomy or laparoscopy.

## 4. Conclusion

A high index of suspicion for parasitic fibroids should be entertained in women presenting with intraabdominal swelling with benign clinical features especially for those with previous history of uterine fibroids. This report also raises questions for further discourse and research on the growth and persistence of uterine fibroids after menopause.

## Supplementary Materials

Supplementary Materials Figure 5 shows the huge parasitic leiomyoma occupying the entire abdominal cavity. Figure 6 shows a huge parasitic leiomyoma.
