# Supplementary material for: Huge Primary Parasitic Leiomyoma in a Postmenopausal Lady: A Rare Presentation
Source: Case Rep Obstet Gynecol. 2019 Apr 1;2019:7683873. doi: 10.1155/2019/7683873 (PMC6463606; doi:10.1155/2019/7683873)
Supplement: Supplementary Materials — Figure 5 shows the huge parasitic leiomyoma occupying the entire abdominal cavity. Figure 6 shows a huge parasitic leiomyoma. [file 7683873.f1.docx]

**SUPPLEMENTARY MATERIALS**

**
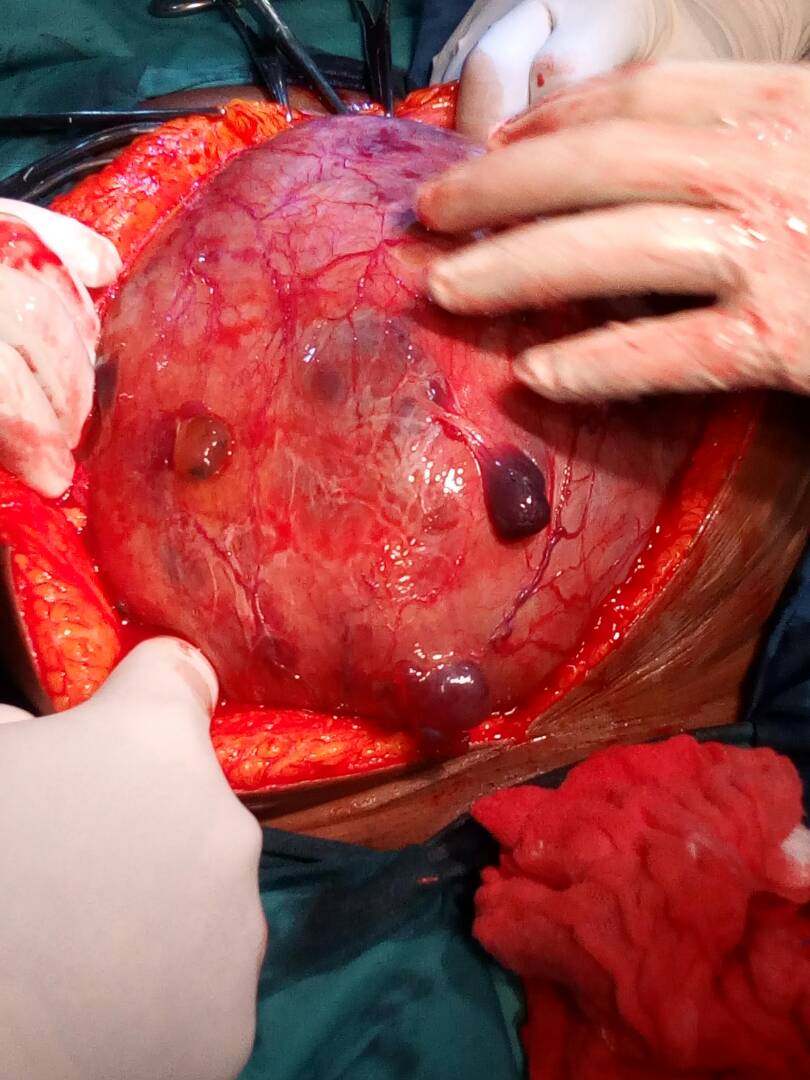
 Fig 5 showing the huge parasitic leimyoma occupying the entire abdominal cavity**

**
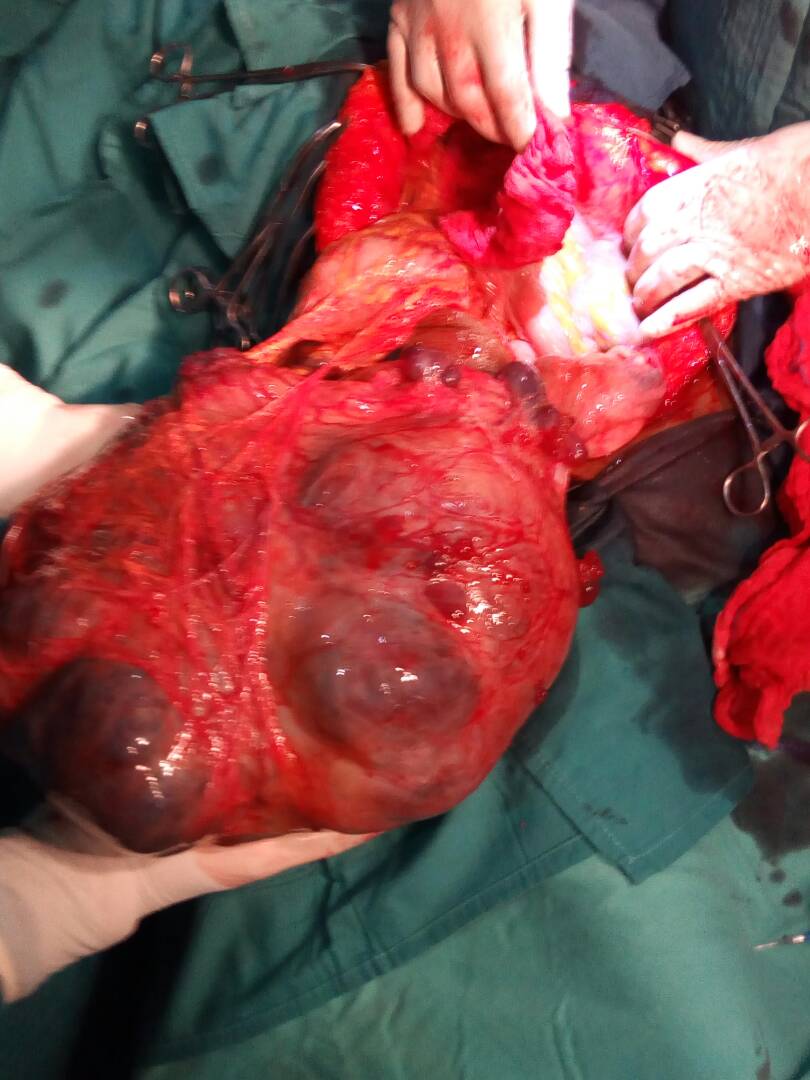
Fig 6 showing a huge parasitic leiomyoma**
